# No evidence for an up-regulation of female immune function in response to elevated risk of sexual conflict

**DOI:** 10.1098/rsbl.2024.0141

**Published:** 2024-09-04

**Authors:** Blake W. Wyber, Joseph L. Tomkins, Leigh W. Simmons

**Affiliations:** ^1^ Centre for Evolutionary Biology, School of Biological Sciences, The University of Western Australia, Crawley 6009, Australia

**Keywords:** phenotypic plasticity, genital damage, immunity, phenoloxidase, *Callosobruchus maculatus*

## Abstract

Sexual conflict is widespread among sexually reproducing organisms. Phenotypic plasticity in female resistance traits has the potential to moderate the harm imposed by males during mating, yet female plasticity has rarely been explored. In this experiment, we investigated whether female seed beetles invest more in immunocompetence, measured as phenoloxidase (PO) capacity, when exposed to cues signalling a greater risk of sexual conflict. Risk perception was manipulated by housing focal individuals alone or with a companion as developing larvae, followed by exposure to a mating-free male- or female-biased social environment when adults. We predicted that females exposed to cues of increased sexual conflict would have increased PO capacity. However, PO capacity did not differ between either larval or adult social treatments. Our results suggest that females may not perceive a risk to their fitness on the basis of increased male presence or are unable to adjust this aspect of their phenotype in response to that risk.

## 1. Introduction

Sexual selection has favoured the evolution of a range of pre- and post-copulatory traits that affect male competitive fertilization success [1,2], and many of these traits can impose significant fitness costs on females [3,4]. Male traits that can be costly to females are diverse in form and can include courtship harassment and harmful genitalia or ejaculate components [5–7]. Female traits that lessen or nullify the impact of harmful male traits are also widespread and diverse, including morphological, physiological and behavioural traits that arise in females due to sexually antagonistic coevolution [8–12]. In theory, phenotypic plasticity in female resistance traits can reduce the likelihood of runaway selection and prevent the coevolutionary arms races that can arise due to sexual conflict [13,14]. However, whether female resistance traits can be plastically adjusted in response to the current risk of male harm remains largely unexplored.

The social environment can elicit phenotypic plasticity in male responses to sperm competition risk, for example in traits such as ejaculate composition, courtship and even genital morphology [15–18]. Plastic adjustments in resistance traits by females, however, remain less well documented. Female guppies (*Poecilia reticulata*) show marked increases in swimming efficiency following exposure to male courtship harassment over a period of five months [19]. Female guppies may thereby invest in increased swimming efficiency to better escape harassing males. Additionally, female house mice (*Mus domesticus*) provided with cues signalling greater sperm competition risk produce ova more resistant to fertilization which may reduce rates of polyspermy under sperm competition [20]. Here, we use a widely studied system in sexual conflict research, the seed beetle *Callosobruchus maculatus*, to examine phenotypic plasticity in a female resistance trait.

In seed beetles, male genital spines damage the female reproductive tract during matings that are typified by females apparently resisting males by kicking [21–23]. Genital wounding improves male fertilization success [24] but comes at a considerable cost to females [5,25]. Wound healing in insects involves the activation of an immunologically active enzyme, phenoloxidase (PO). PO activation is costly because it increases metabolic rate by up to 28%, is associated with a reduced lytic activity which would otherwise protect the insect against bacterial pathogens [26] and can reduce lifespan [27]. Given the fitness costs of immune activity, female investment in PO activity should depend on both the evolutionary history and immediate risks of immunological challenges [28,29]. In *C. maculatus,* there is substantial among-population variation in PO activity that is positively associated with the intensity of sexual conflict [11]. Moreover, female *C. maculatus* evolving in the absence of sexual conflict show reduced PO capacity, and there has been a positive correlated evolution of PO capacity and the harmfulness of male genitalia across species of seed beetles generally [30]. Here, we ask whether female seed beetles can plastically adjust their PO capacity in relation to the degree of harm they expect to encounter in their current mating environment. We predicted that females exposed to a male-biased social environment should increase their capacity to mount a PO response in anticipation of elevated reproductive tract damage.

## 2. Methods

### (a) Stock population

Individuals were sourced from a population of seed beetles maintained at 26°C and subject to a 12 L : 12 D cycle. The population was founded in 2005 from individuals obtained from the CSIRO in Canberra, ACT. Populations of >500 beetles were reared on *ca* 250 cm^3^ mung beans (*Vigna radiata*) for approximately 10 generations prior to use in experiments.

### (b) Larval social environment

A container of approximately 500 mung beans was provided to the single stock population for a period of one week. Beans were inspected daily, and those with a single egg were sampled for use in the experiment. For logistical reasons, the experiment was conducted in two blocks.

Beans were sorted evenly into one of two treatments, representing the presence or absence of larval competition. In the larval competition treatment, two beans each with a single egg were packed tightly together using a plug of cotton wool within a ventilated 1.5 ml Eppendorf tube. Larvae of this species can detect vibrations from conspecifics within neighbouring beans with effects on growth and development [31], so this manipulation aimed to facilitate social signals of future competitors and/or mates. The no-competition treatment had focal beans with a single egg packed tightly with an uninfested bean. Approximately three weeks later, beans were inspected daily for ‘windows’ on the focal beans. The appearance of a window indicates that individuals are approximately one week from emerging. To prevent individuals within the larval competition treatment from interacting and/or mating, both beans were isolated at the window stage in separate Eppendorf tubes. Tubes were inspected daily for emerging females. In the larval competition treatment, the first female to emerge from either of the beans was considered the focal individual, and the sex of their companion, once emerged, was recorded. Following emergence, each focal female was weighed using a *Sartorius SE3* micro-balance.

### (c) Adult social environment

**Figure 1.**
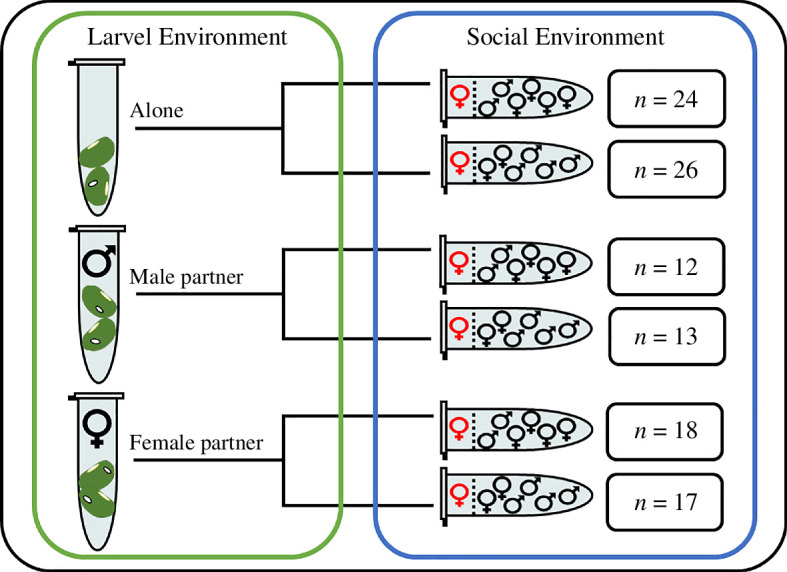
Experimental design. Focal female individuals developed either alone in an Eppendorf tube or with another developing larvae in a separate mung bean, that was sexed post-emergence. Individuals were then exposed to either a male or female biased social environment over 7 days.

Emerged females from each larval competition treatment were assigned randomly to one of two adult social environments that reflected either a high or low risk of sexual conflict. In the high-risk treatment, focal females were placed within the lid of a 1.5 ml Eppendorf tube separated from four males and two females by a fabric mesh. Individuals assigned to the low-risk treatment were separated from two males and four females. This experimental protocol has been used elsewhere to elicit plastic adjustments in male ejaculate allocation patterns and female kicking behaviour [21,22,32]. Thus, we are confident that females within the present study should have perceived cues of sufficient strength to induce plasticity. Focal females were exposed to their respective social environments for a period of one week before being weighed and assayed for PO capacity. In total, 110 females were analysed (see figure 1): 31 in block 1 and 79 in block 2.

### (d) Phenoloxidase extraction and analysis

The capacity to mount a PO response typically decreases immediately following mating, presumably because pro-PO is converted to PO to affect wound healing but returns to pre-mating baseline levels 1–2 days following mating [30]. To allow direct comparison with previous studies, we measured the maximum PO capacity from whole-body extracts of unmated females [11,30]. PO assays followed the methodology outlined by Bagchi *et al*. [30]. Whole beetles were crushed in 20 µl phosphate-buffered saline (PBS) in 1.5 ml Eppendorf tubes and centrifuged at 21 130 rcf for 10 min at 0°C. Subsequently, 10 µl of supernatant was removed and stored at −80°C for approximately 24 h to ensure all pro-PO had converted to PO [30].

After the homogenate had thawed, each sample was agitated manually and then centrifuged for approximately 30 s. A 3 µl aliquot of the homogenate was taken and combined with 7 µl PBS and 50 µl of 10 mM dopamine solution. Additionally, a blank for each sample was created in which the dopamine solution was replaced with 50 µl of Millipore water. Both blanks and the samples were distributed across the wells of 96-well microlitre plates. Microlitre plates were analysed using an M5 *Spectramax* Plate reader at 25°C, with absorbance measured at 420 nm every 30 s for 15 min. PO activity (Δ420 nm min^−1^) of each sample was corrected by removing the absorbance rate of its corresponding blank.

### (e) Statistical analysis

Statistical analysis was conducted in R (v. 4.0.3) R Development Core Team 2016. Linear models were run using the package *lme4* [33] to test the significance of the experimental treatments on PO capacity. Models with and without an interaction term between adult and larval social environment were compared using AICc, with both found to be within two AIC values. Thus, the most complex model was selected to ensure conservative estimates of the effect of all treatments. Larval environment was included as a fixed effect, with levels reflecting individuals experiencing no larval competition, a female larval partner or a male larval partner. Similarly, adult social environment was included as a fixed effect, reflecting either male- or female-biased manipulations. Female eclosion weight was included as a covariate within the model to account for any variance in PO capacity due to body size. Block was included as a fixed effect, rather than as a two-level random effect, to avoid model overfitting. We calculated Cohen’s *f* ^2^ to evaluate the strength of individual effects within the context of our multivariate models.

## 3. Results

**Figure 2.**
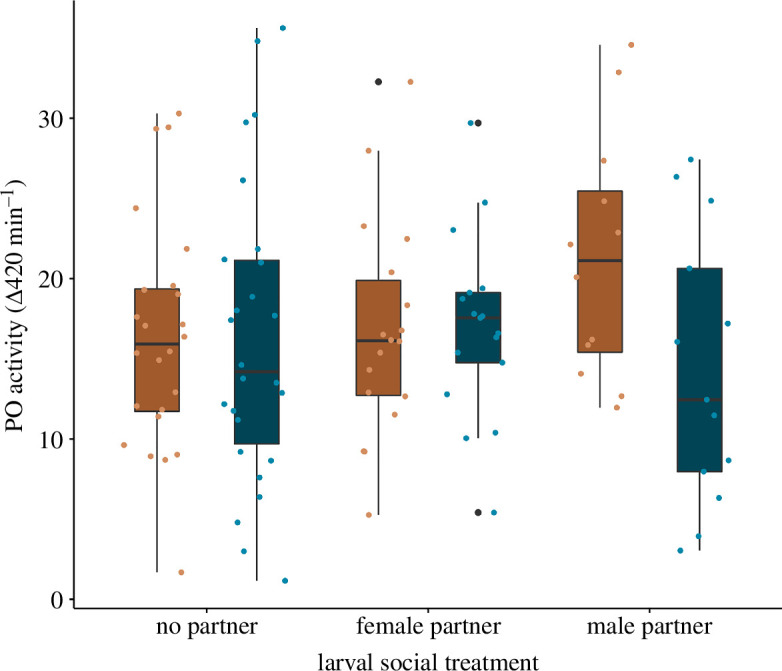
Boxplot of the effects of female larval and adult social environments on constitutive phenoloxidase capacity. Females that developed as larvae alone, with a female or with a male in the adjacent bean were subsequently exposed to either a female-biased (brown/pale) or a male-biased (blue/dark) social environment as adults. Boxes represent the upper and lower quartiles, the line represents the median and the whiskers represent the upper and lower extremes, excluding potential outliers which are indicated as black circles.

There were no significant interaction effects or main effects of either larval or adult social environment on the constitutive PO capacity of female seed beetles (table 1; figure 2). Experimental block explained little variance in the data. Model residuals were normally distributed (Shapiro–Wilks, *p* = 0.059) and variances were homoscedastic (Bartlett test, *p* = 0.402) (see code in electronic supplementary material).

**Table 1.** ANOVA, with effect sizes and associated 95% confidence intervals of the linear mixed model of larval rearing and adult social exposure on levels of female phenoloxidase activity.

	*f*-value	d.f.	*p*‐value	Cohen’s *f* ^2^	95% CI
intercept	35.89	1, 102	<0.001		
adult social environment	0.009	1, 102	0.92	0.01	0.00–0.09
larval social environment	1.85	2, 102	0.16	<0.01	0.00–0.05
female weight	1.08	1, 102	0.30	0.01	0.00–0.09
block	0.07	1, 102	0.79	<0.01	0.00–0.05
adult × larval social environment	2.12	2, 102	0.12	0.04	0.00–0.14

## 4. Discussion

We predicted that female seed beetles exposed to cues signalling a greater risk of genital wounding, whether from cues perceived as larvae and/or as adults, would upregulate their PO capacity in anticipation of the demands for wound healing. However, we found that females who developed with a conspecific had similar PO capacity to females who developed alone, and females exposed to a male-biased social environment had comparable PO capacity to females exposed to a female-biased social environment. These data suggest that either our treatments were ineffective in manipulating the social cues necessary for females to plastically adjust their expenditure on wound healing, or they are unable to make anticipatory adjustments in PO capacity.

Previous studies of *C. maculatus* have found evidence of correlated evolution in female immune function, both in response to the severity of male genital damage and population skew in sex ratio [11,32]. Dougherty *et al*. [11] found that the elaboration of male spines and female PO capacity were correlated among 13 discrete populations of seed beetles. This micro-evolutionary pattern suggests that female constitutive immunity can coevolve with male-induced genital harm among populations of *C. maculatus*. Comparative analysis of PO capacity and male penile spine length across the genus also shows a similar macro-evolutionary pattern, where species with more injurious male genital morphology have greater PO capacity in females [30].

While PO capacity may evolve in response to elevated sexual conflict among populations, we found no evidence to suggest that plasticity within a generation can mitigate the risk of male-induced harm. Apart from the present study, a single study has sought to identify phenotypic plasticity in PO capacity in seed beetles [30]. Bagchi *et al*. [30] manipulated female mating history by providing females the opportunity to mate once per day across three consecutive days. Females that copulated more frequently had markedly reduced PO capacity compared to those that remain unmated. However, in that study, females were assayed for PO at the end of the third day, and females that mated once on the final day had comparable PO capacity to those that mated on all 3 days. Thus, the patterns observed could arise from PO depletion following recent wound healing, rather than because of phenotypic plasticity in female expenditure.

An explanation for why individuals may not have responded plastically in the present study may be that our treatments were ineffective in manipulating social cues or that the critical period for plasticity was missed. Our method for manipulating the adult sex ratio has been shown numerous times to affect plasticity in reproductive behaviour [21,22,32]. In contrast, our manipulation of the larval social environment was based on just one previous study of competitive growth [31]. Moreover, to ensure females remained unmated on emergence, in our larval manipulations, we removed paired beans at the onset of ‘windows’ appearing on beans, when seed beetles undergo pupation. Should the pupal stage of development be critical for plasticity in PO capacity, the absence of competition cues in this period may explain our results. However, PO capacity seems to be extremely low during larval and pupal development and massively upregulated in adult females [30], the developmental stage where our method of social manipulation has proven effects on both male and female reproduction.

Finally, to be directly comparable with micro- and macro-evolutionary studies of female immunological responses to variation in sexual conflict intensity, we measured the whole-body PO capacity of unmated females [11,30]. It may be that females show plasticity in a localized, tissue-specific response in PO capacity, or in the rates at which they can replenish pro-PO following mating. Such abilities have not yet been studied in *C. maculatus*. However, our finding of a lack of plasticity in whole-body PO capacity in response to cues to sexual conflict is consistent with the findings of Wyber *et al*. [34]. They found no evidence for plastic changes in female reproductive tract thickness in response to cues signalling greater risk of sexual conflict, even though reproductive tract thickness varies in response to sexual conflict intensity both among populations of *C. maculatus* and across seed beetle species generally [11,35]. Collectively, our studies offer little support for the notion that female seed beetles adjust their resistance to male harm in response to the immediate sociosexual environment.

In conclusion, although there is evidence of evolutionary responses in female seed beetles to sexual conflict at both macro- [30] and micro-evolutionary [11] scales, we found no evidence that female *C. maculatus* can elevate their investment in wound healing when exposed to locally available cues of sexual conflict risk. Our findings suggest that a fixed allocation to female resistance may be adaptive, perhaps because female seed beetles experience little within-population variation in male harm. Nevertheless, female plasticity holds important theoretical implications [13,14], warranting further exploration of phenotypic plasticity in other systems where sexual conflict has been identified.

## Data Availability

The data and R code required to reproduce the analyses reported in this paper are available in the electronic supplementary material [36].
